# Tracking of Endothelial Cell Migration and Stiffness Measurements Reveal the Role of Cytoskeletal Dynamics

**DOI:** 10.3390/ijms23010568

**Published:** 2022-01-05

**Authors:** Dominick J. Romano, Jesus M. Gomez-Salinero, Zoran Šunić, Antonio Checco, Sina Y. Rabbany

**Affiliations:** 1 Bioengineering Program, DeMatteis School of Engineering and Applied Science, Hofstra University, Hempstead, NY 11549, USA; dromano2@pride.hofstra.edu (D.J.R.); achecco@veeco.com (A.C.); 2 Department of Medicine, Weill Cornell Medical College, New York, NY 10065, USA; jmg2008@med.cornell.edu; 3 Department of Mathematics, Hofstra University, Hempstead, NY 11549, USA; Zoran.Sunic@hofstra.edu

**Keywords:** atomic force microscopy, cell migration modeling, biased persistent random walk, turning angle distribution, biomechanics, sheet migration, cell elasticity, time-lapse imaging

## Abstract

Cell migration is a complex, tightly regulated multistep process in which cytoskeletal reorganization and focal adhesion redistribution play a central role. Core to both individual and collective migration is the persistent random walk, which is characterized by random force generation and resistance to directional change. We first discuss a model that describes the stochastic movement of ECs and characterizes EC persistence in wound healing. To that end, we pharmacologically disrupted cytoskeletal dynamics, cytochalasin D for actin and nocodazole for tubulin, to understand its contributions to cell morphology, stiffness, and motility. As such, the use of Atomic Force Microscopy (AFM) enabled us to probe the topography and stiffness of ECs, while time lapse microscopy provided observations in wound healing models. Our results suggest that actin and tubulin dynamics contribute to EC shape, compressive moduli, and directional organization in collective migration. Insights from the model and time lapse experiment suggest that EC speed and persistence are directionally organized in wound healing. Pharmacological disruptions suggest that actin and tubulin dynamics play a role in collective migration. Current insights from both the model and experiment represent an important step in understanding the biomechanics of EC migration as a therapeutic target.

## 1. Introduction

Blood vessel formation is carried out by endothelial cell (EC) migration, and changes in cell morphology. Most ECs form the inner structure of the vascular system and are quiescent with a regular cobblestone appearance and little migratory and proliferative activity. ECs change into an activated phenotype and start to migrate and proliferate to form new vessels in response to nutritional and oxygen demands within a tissue, in a process termed angiogenesis. Angiogenesis and vascular remodeling are driven by EC capacity for collective motility and coordination with surrounding ECs [1]. A key building block to collective motility is individual EC movement, which manifests itself in two forms: (1) Random motility—EC directional preference is uniform in time and space (isotropic); and (2) directed motility—EC directional isotropy is broken in favor of a particular direction (anisotropic), driven by external stimuli such as physical and chemical cues [2,3,4]. In any case, the migrating cell has an asymmetric morphology with a leading edge that forms membrane protrusions, and a trailing edge where its adhesions are locally removed. The regulation of EC adhesion and motility is critical for vascular remodeling, much of which is carried out by filopodia and lamellipodia [2,5,6,7]. Over the entire process, actin and tubulin crosstalk play a vital role in coordinating the network reorganization needed to facilitate migration.

Along with coordinating cell motility, the cytoskeleton is responsible for several vital cell processes including adhesion, morphology, and structural integrity. Previous studies on 3T3 and NRK fibroblasts found that the actin cytoskeleton plays a role in maintaining the cell’s Young’s modulus while the tubulin cytoskeleton does not affect the elasticity of the cell [8]. More recent studies have investigated the role of tubulin dynamics in HeLa, L929, and fixed human umbilical vein endothelial cells (HUVECs) [9,10,11]. We propose that EC elasticity also contributes to EC migration. To elucidate the role of cytoskeletal dynamics on stiffness and migration, we utilized Atomic Force Microscopy (AFM), cytoskeletal disruptions—cytochalasin D (CD) for actin and nocodazole (Noc) for tubulin—on confluent EC monolayers and wound healing models. Our observations suggest that disrupted actin and tubulin dynamics lowers the compressive modulus of ECs over time. Time-lapse data show a significant reduction in gap closing speed for actin and tubulin-disrupted ECs, suggesting a link between cytoskeletal dynamics, stiffness, and EC migration. Earlier studies proposed predictive mathematical models [12,13,14] aim to describe and predict EC motility. Previous models of cell migration involve a random process for force generation, a persistence term to represent resistance to vector changes in velocity, and a directional bias term to account for an external stimulus [14]. We utilize a framework based on persistent random walks as a tool to describe the distribution of the turning angle of cell movement depending on its current direction. The model and measurements from time-lapsed EC wound healing assays demonstrated that EC persistence is not uniform over all directions, and that EC speed is regulated directionally in an in vitro wound (for the remainder of this work, an in vitro scratch assay will be referred to as a “wound”). We found that ECs persistence and speed are greatest in the direction toward the wound gap, smaller in the lateral direction to the gap, and slightest in the direction away from the gap, suggesting that cell–cell crosstalk may play a role in regulating cell speed and persistence during sheet migration. When looking at the cytoskeletal disruptions, the measured persistence was lower compared to the controls, indicating that cytoskeletal dynamics contributes to EC persistence. 

## 2. Biased Persistent Random Walk Model of Cell Migration

We model the EC movement by a biased persistent random walk, also known as a biased correlated random walk [15]. In this model, it is the change in the magnitude and direction, or vector subtraction, of the velocity vector (from one timepoint to the next) that is considered a discrete random process. Such processes have been used extensively in biology, and are known as velocity jump processes [16]. There is no universally adopted definition of persistence, but it is generally understood that it represents the tendency of a cell to maintain its current velocity or, in particular, its current direction of movement. One way to measure this tendency is to look at the correlation between the successive directions of movement [17]. In any model based on extensions or adaptations of the Uhlenbeck–Ornstein process [18], that is, any model in which the velocity change $d\vec{v}\;$involves a component of the form $\beta \;\vec{v}dt$ proportional to the current velocity, the persistence is understood as the time needed for the influence of the current velocity to completely dissipate, and this time is given by 1/β. Note that the Uhlenbeck–Ornstein process is an extension of the Wiener process (modeling Brownian motion) obtained by introducing the persistence component $\beta \vec{v}dt$.

A straightforward way of measuring the persistence of a cell movement is to consider its mean square displacement function MSD(t) (average, taken for all cells, of the square of the total displacement of the cell between time 0 and time t). When the cell movement is unbiased it is completely random, without any persistence. That is, when there is no relation between the consecutive velocity vectors, the movement is diffusive and the MSD function is linear with time. Conversely, if all cells move along straight lines, exhibiting maximal possible persistence, the displacement function is quadratic. In practice, neither of these extremes occurs for cell movement and the MSD function is modeled by a power law $MSD\left(t\right)∼t^{\mu }$, where 1≤μ≤2. The coefficient μ can easily be estimated by linear regression applied to the log-log transformed data for MSD and time. The larger values of μ correspond to higher persistence.

**Figure 1:**
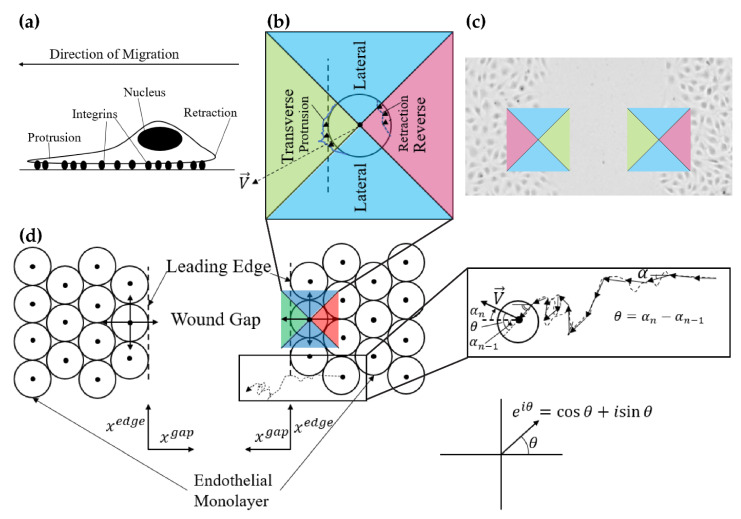
(**a**) Simple schematic detailing endothelial cell migration at the cellular level; (**b**) Visual representation of the migration zone convention is denoted by: Transverse (across the gap), lateral (generally parallel to the leading edge), and reverse (opposite to the gap) directions in the context of the leading edge of a wound. In healing, cell transverse velocities are ideal (moving across the gap), the reverse region is antithetical to wound healing, and velocities in the lateral region are neither as bad as the reverse region nor as good as the transverse region; (**c**) Migration zones in the context of a wound healing assay; (**d**) A simplified model of a wound healing assay. In this view, one may more clearly see the transverse, lateral, and reverse zones in relation to the leading edge. A representative single EC motion is identified, and we denote the equation for the turning angle θ. Note that the sign of the angle is important (counterclockwise form the horizontal is positive). Lastly, we show the representation of a two-dimensional vector as a polar complex number, which was used for brevity throughout the computations.

In our case, the cell movement is anisotropic, meaning that there is a preferred direction of movement. In a wound, the preferred direction of movement is toward the wound; hence, our random walk is not only persistent but also biased. The presence of a bias is easily established, the mean displacement in the direction parallel to the wound edge is, in some of our experiments, less than half of the mean displacement in the direction orthogonal to the wound. We go into a deeper analysis of the anisotropy by considering the distribution of the turn angle for various groups of cells, based on their current direction of movement. As seen in Figure 1, the angle measured in the positive direction (counterclockwise) from the *x*-axis at step n is $\alpha_{n}$, its turning angle $\theta_{n}$ is defined as

(1) $$\theta_{n}=\alpha_{n+1}-\alpha_{n}$$

where the representative angle satisfies $-\pi \leq \theta_{n}\leq \pi$. The turning angle distribution is related to the persistence through the concentration around θ=0. More persistent migration will approach a high concentration of turning angles around 0. Three circular distributions are used in the literature to model turn angles: von Mises, wrapped normal, and wrapped Cauchy [15]. However, the first two have much thinner tails than the empirical distributions obtained from the experimental data and we model the turn-angles by using the wrapped Cauchy (also known as the wrapped Lorentzian) distribution with mean 0 and scale parameter ρ, whose probability density function is

(2) $$f\left(\theta \right)=\frac{1}{2\pi }\left(\frac{1-\rho^{2}}{1+\rho^{2}-2\rho \cos\left(\theta \right)}\right).\;$$

The scale parameter ρ varies from 0 to 1, and 0 corresponds to uniform distribution (thus the choice of a turn angle that is uniformly random, that is, no persistence at all) and 1, in the limit, to Dirac distribution centered at θ=0 (thus, no change in the turn angle, that is, maximal possible persistence). We take the parameter ρ as the measure of cell persistence since higher values of ρ correspond to a higher concentration of turn angles around 0 and, thus, to a higher tendency of maintaining the current direction. Better yet, the wrapped Cauchy distribution fit the peak and tails of all empirical histograms well.

A quick estimate of the parameter ρ can be obtained by the magnitude of the average turn-angle vector $e^{i\theta }$ (the average taken over all cells and all steps), but this estimate is biased. Instead, we use the maximum likelihood estimate (MLE) of Kent and Tayler [19].

Note that the distribution of the turn angles depends on the choice of the time interval used to measure the turn angles and, as such, the estimate of the parameter ρ is not very useful in an absolute sense. However, in all our experiments the time interval is the same, 180 s, and we are only using the estimates of ρ to make comparisons of cell persistence in various directions and between cells subject to different treatments, but within the same biological replicate. Along with the prescribed persistence analysis, collective wound healing can be meaningfully summarized with the mean coordinate displacement and total displacement. Given the position vector x at time $t_{0}\leq t\leq t_{f}$ for the $k^{th}$ cell, $x_{k}\left(t\right),\;t\in \left[t_{0},t_{f}\right]$, the displacement vector between positions $x_{k}\left(t_{0}\right)$ and $x_{k}\left(t_{f}\right)$ is defined,

(3) $$u_{k}=x_{k}\left(t_{f}\right)-x_{k}\left(t_{0}\right)$$

The mean coordinate displacement looks to compare transverse (optimal), absolute lateral, and reverse (worst) displacements for all cells. That is, we can split the displacement components along two axes, $u^{x}$: Lateral axis; $u^{y}$: Transverse-reverse axis (Figure 1). We then separate the coordinate displacements for the $i^{th}$ cell:

$$\mathrm{Transverse}:\;u_{i}^{T}=\left|u_{i}^{y}\right|,\;\mathrm{when}\;u_{i}^{y}>0$$

$$\mathrm{Lateral}:\;u_{i}^{L}=\left|u_{i}^{x}\right|$$

$$\mathrm{Reverse}:\;u_{i}^{R}=\left|u_{i}^{y}\right|,\;\mathrm{when}\;u_{i}^{y}<0.$$

We only compared $u_{i}^{T}$ and $u_{i}^{L}$ for transverse-displaced cells because the sample size of reverse-displaced cells was too small for accurate comparisons. Note that the x and y displacement are coupled for each cell which requires a paired test for comparison. For the $N_{T}$ transverse displaced cells, we obtain our mean transverse and lateral coordinate displacements:

(4a) $$\begin{array}{c} \langle u^{T}\rangle =\frac{1}{N_{T}}\overset{N_{T}}{\underset{k=1}{\sum }}u_{k}^{T}\; \end{array}$$

(4b) $$\begin{array}{c} \langle u^{L}\rangle =\frac{1}{N_{T}}\overset{N_{T}}{\underset{k=1}{\sum }}u_{k}^{L} \end{array}$$

To obtain the mean total displacement, we first need the displacement magnitude. The total (scalar) displacement is defined from the displacement vector magnitude for the $k^{th}$ cell,

(5) $$u_{k}\equiv ∥u_{k}∥=\sqrt{{\left(u_{k}^{x}\right)}^{2}+{\left(u_{k}^{y}\right)}^{2}}$$

The mean total displacement over N cells is,

(6) $$\langle u\rangle =\frac{1}{N}\overset{N}{\underset{k=1}{\sum }}u_{k}.\;$$

Equations (5) and (6) will be used to compare the migratory behavior wound healing ECs in the conducted experiments.

## 3. Results

### 3.1. Cytoskeletal Dynamics Contributes to Wound Healing and Gap Closing Speed

**Figure 2:**
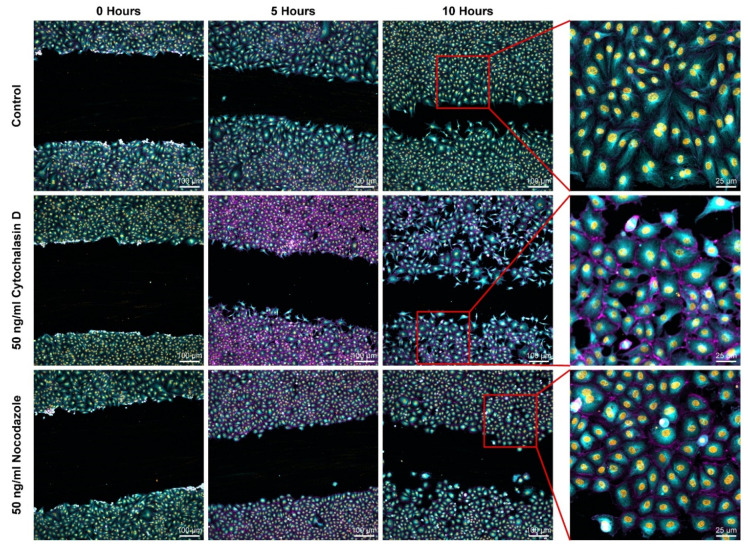
Wound healing assays for cytoskeletal disruptions and control. Stained actin is presented in purple, tubulin stains are shown in green, and then nuclear counterstain is provided in yellow. To better view single cell morphology, 4X zoom-in of the field of view is provided for each condition.

**Figure 3:**
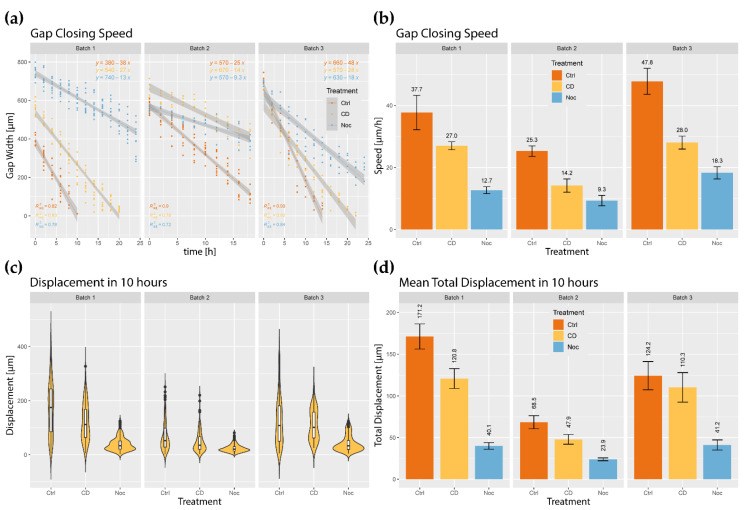
(**a**) Linear regressions of collected gap distance data at increasing time points for cytoskeletal disruptions (Ctrl—control; CD—50 ng/mL cytochalasin D; Noc—50 ng/mL nocodazole). The slope of the linear regression is the gap closing speed; (**b**) Gap closing speeds comparing cytoskeletal disruptions, with the bars representing the respective 95% confidence interval; (**c**) Violin plot of EC 10-h displacements under different cytoskeletal disruptions in a wound healing assay; (**d**) Mean displacement in 10 h. The error bars correspond to the 95% confidence interval.

Time lapses of wound healing assays under various conditions are used throughout this work to describe the effects of inhibited dynamics on cell migration. To probe these effects on a large scale, live stains of actin, tubulin, and nuclei of HUVEC wound healing models were prepared according to pharmacological disruptions (Figure 2). Actin and tubulin-disrupted monolayers appeared to close the wound slower than the control condition (Figure 2). To investigate this further, the gap closing speeds of wound healing time lapse assays were measured (Figure 3a,b). Actin-disrupted ECs closed the wound as little as half as fast compared to the control, with a minimum reduction of 28% in gap closing speed compared to the control (Figure 3b). Likewise, tubulin disruptions lower gap closing speeds by 62% to 66% compared to the control (Figure 3b).

Tying in the large-scale behavior of gap closing speed and individual cell migration, the box and violin plots present the displacement distribution of ECs (Figure 3). Control and actin-disrupted wound healing models displayed a higher maximum displacement, while tubulin-disrupted monolayers had a bias toward no displacement. From the distribution, the mean total displacement was calculated. Actin-disrupted cells produced significantly lower mean total displacement compared to the control, whereas tubulin-disrupted monolayers displayed a decrease in mean displacement compared to the control monolayers (Figure 3d).

From both the full field of view and 4X zoom-in, qualitative differences in cell morphology can be identified (Figure 2). Control HUVECs appear oblate with clear definition in their stress fibers. Notably, the control monolayers appear simply connected, meaning that no holes are found in the gap-separated monolayers (Figure 2). After disrupting actin polymerization, irregular morphology can be identified, and the existing actin appears concentrated at the cell periphery (Figure 2). At the 10-h mark, the actin-disrupted monolayer is no longer simply connected—numerous instances of cell–cell connectivity loss are identified (Figure 2). Tubulin-disrupted cells appear with more subtle changes. Firstly, the individual ECs appear less oblate, and instances of cell connectivity loss are identified (Figure 2).

### 3.2. ECs Organize Speed and Persistence in a Directionally Dependent Manner

To probe the behavior of ECs in wound healing, we split all possible directions of EC migration into four equally spaced regions: One transverse, two lateral, and one reverse region as shown in (Figure 1). We are interested in the ability of the cell to behave differently depending on the instantaneous direction in which it migrates. If the cells have such ability, we say that they are directionally organized in speed, persistence, etc.

**Figure 4:**
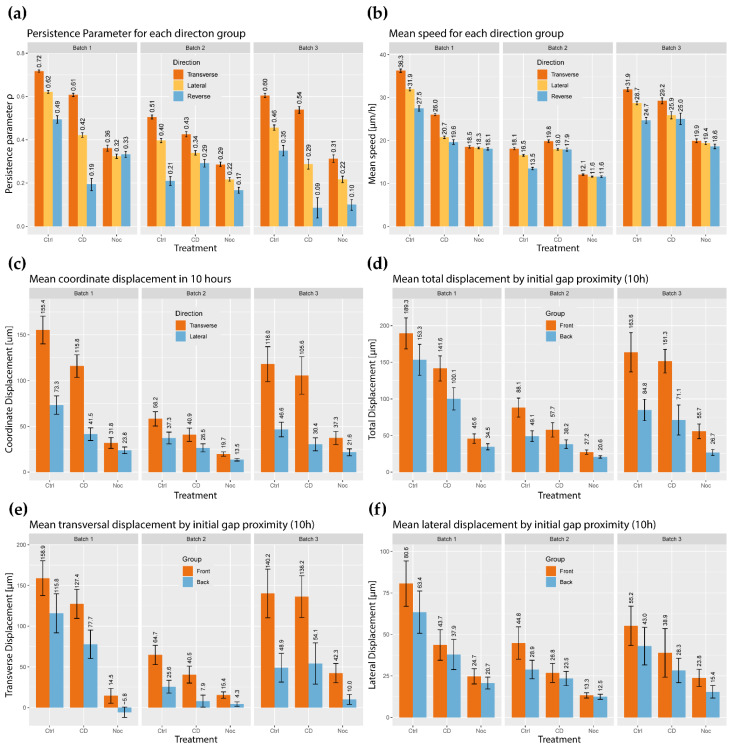
(**a**) Persistence parameter for each direction group; (**b**) Mean speed calculated for motion in each direction group; (**c**) Mean coordinate displacement in 10 h by direction grouping. The reverse direction was excluded from the visualization due to few samples (see Supplementary Materials); (**d**) Mean total displacement in 10 h by initial proximity to the wound gap; (**e**) Mean transversal (orthogonal to the leading edge) displacement in 10 h by initial proximity to the wound gap. As described in Figure 1, the ideal motion for wound healing is positive coordinate displacement along the transverse axis; (**f**) Mean coordinate displacement of the lateral component (parallel to the leading edge) in 10 h by initial proximity to the wound gap. The error bars represent the 95% confidence interval. Definitions for the displacement and total displacement can be found in the framework section.

The turning angles of cell tracks calculated from Equation (1) form a distribution for each experiment. Combined with the migratory region definition, the turning angles may be further sorted into directional groups. Due to the nature of the turning angle distribution, persistence was defined as the MLE of the scale parameter in Equation (2), denoted as ρ (Figure 4a). As a note, ρ is dependent on the imaging interval, so it is not an absolute measure of persistence; however, this MLE is good for comparing between regions and experimental groups. When looking at the MLEs, the control ECs in a wound were most persistent in the transverse region, less persistent in the lateral regions, and least persistent in the reverse region (Figure 4a). This result suggests that ECs in wound healing are most resistant to directional change when moving in the optimal direction, and more readily turn when off course. Actin-disrupted monolayers were less persistent in all directions compared to the control yet managed to directionally organize their persistence (Figure 4a). After nocodazole treatments, all three regions were less persistent than the control, but the directional organization of persistence was preserved (Figure 4a).

The next step was to see if EC speed was sensitive to direction in wound healing, and as such the mean speed was computed for each directional grouping. In the controls EC speed was highest in the transverse direction, lower in the lateral zone, and lowest in the reverse region, suggesting that EC speed is sensitive to direction in wound healing models (Figure 4b). In the actin-disrupted group, ECs maintained higher mean speeds in the transverse region than in both the lateral and reverse region; however, there was no discernible difference in measured speeds between the lateral and reverse regions (Figure 4b). There was no noticeable speed sensitivity to a direction in the tubulin-disrupted monolayers (Figure 4b). Therefore, EC migration speed and directionality are primarily affected by tubulin dynamics as well as actin dynamics to a lesser extent.

Following up with the results from the mean speed, the mean coordinate displacement in 10 h was calculated for each experiment by directional grouping. For the purposes of displacement calculations and comparisons, the cells in each assay were divided into two types—those that got closer to the gap (transverse cells) and those that got further (reverse cells). As discussed in Section 2, the sample size of the reverse-displaced cells was too small for adequate comparison and thus was excluded from this analysis. For the transverse cells, the mean displacement in the transverse direction was calculated and compared to the mean displacement of the same cells in the Lateral direction. In all cases, displacement across the gap was the highest (Figure 4c). Compared to the control, actin-disrupted wounds appeared to maintain slightly lower transverse mean coordinate displacements compared to the control. Tubulin-disrupted monolayers experienced much smaller mean transversal displacement, and the difference between transverse and lateral, mean coordinate displacement was not as striking when compared with the control monolayers (Figure 4c).

**Figure 5:**
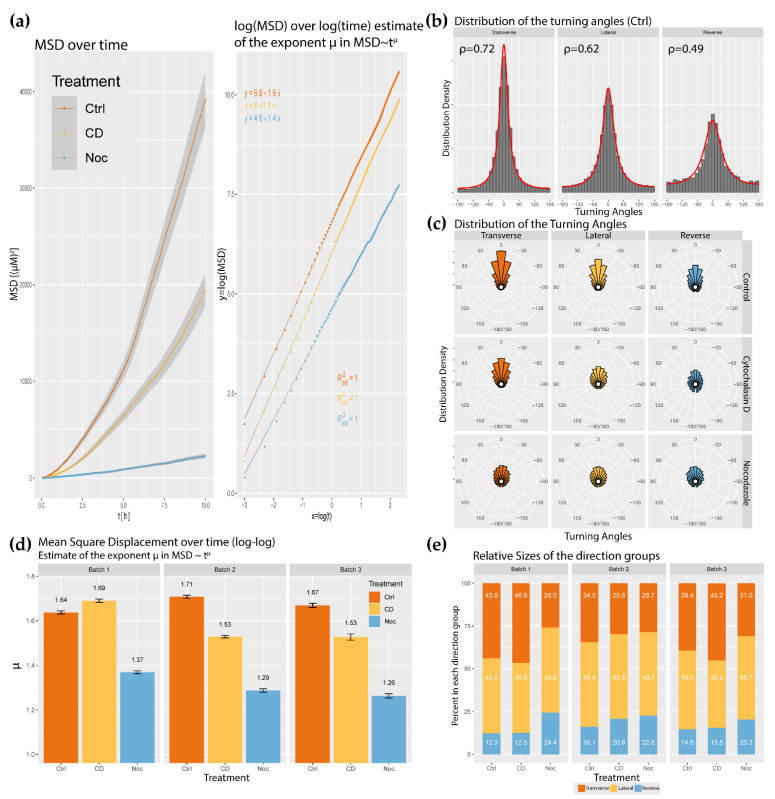
(**a**) Plot of the mean squared displacement (MSD) over time for an ensemble of tracked ECs (*n* = 269). The same plot is transformed into log-log coordinates to estimate the exponent μ in $MSD=at^{\mu }$ with a linear regression. Note that the log(x) function in these calculations refers to the natural logarithm (base e). Note that the standard linear regression problem utilizes the line slope and y-intercept to minimize the error in the dataset; (**b**) Normalized histogram of empirically measured turning angles and the wrapped Cauchy probability density. This was performed on the previously denoted directional regions for cytoskeletal disruptions (Ctrl—control; CD—50 ng/mL cytochalasin D; Noc—50 ng/mL nocodazole). The number on the top left-hand corner represents the maximal likelihood estimate of the concentration around $0^{{}^{\circ}}$ parameter ρ to produce the observed turning angle distributions; (**c**) Histograms from (**b**) represented radially. Positive angles correspond to counter-clockwise rotations from the 0 axis, consistent with the convention used in (**b**); (**d**) Estimated exponent μ in $\mathrm{MSD}\sim t^{\mu }$ comparison by biological replicate (error bars correspond to the 95% confidence interval). (**e**) Relative sizes direction groups for each wound healing assay and cytoskeletal condition.

Continuing with cell displacement, the cells were split into “front” (consists of cells whose initial distance to the leading edge is smaller than the median of all distances to the leading edge) and “back” groups (consists of cells whose initial distance to the leading edge is larger than the median of all distances to the leading edge). This was performed to uncover if there was a general organization of migratory behavior to leading edge proximity. A simple summary of displacement was carried out with the mean total displacement. The control total displacement was generally the highest, followed by actin disruptions and tubulin disruptions. All front grouped cells experienced a larger total displacement in 10 h compared to the back group (Figure 4d). Combining cell proximity with directional organization, the mean transversal displacement (Figure 4e) and the mean lateral displacement (Figure 4f) are plotted with respect to the “front” and “back” definitions. The first is the mean coordinate displacement and its calculation can be found in Section 2. The control monolayers accumulated twice as much displacement orthogonal to the gap as they did lateral to the gap (Figure 4e,f). Actin-disrupted ECs maintained predominantly transverse displacement, although its mean coordinate displacement was not generally as high as that of the controls (Figure 4e,f). Predominant transverse displacement was not as strong in the tubulin-disrupted ECs, and its mean coordinate displacements were lower on all accounts (Figure 4e,f). When looking at the front and back groups, no significant difference was found between the front and back lateral displacement (Figure 4e,f). Throughout the entire observation set, the transversal displacement across the gap was significantly higher in the front ECs compared to those in the back (Figure 4e,f). Returning to persistence, another good summary is the power of μ estimated in $MSD∼t^{\mu }$ for the total mean squared displacements for a sample of ECs in each experiment, as shown in (Figure 5a). To compliment the estimations μ,ρ, the velocity vector turning angle plots are provided (Figure 5b,c). For all observations, the distribution of turning angles is symmetric for all three zones (Figure 5b,c). Furthermore, the normalized histogram of turning angles approaches a wrapped Cauchy distribution of angles from $-180^{{}^{\circ}}<\Theta <180^{{}^{\circ}}$ (Figure 5b,c).

For overall MSD, control ECs generally displayed the highest estimated exponent for the wound healing assay (>1.64), actin-disrupted wounds generally displayed a lower estimated exponent (<1.69) tubulin-disrupted wounds were measured to have a lower estimated exponent compared to the control (Figure 5d). These basic persistence estimates already suggest that in most cases, control ECs are the most persistent, hence the most resistant to directional change. Actin and tubulin-disrupted ECs displayed lower overall persistence when compared to the control.

Calculating the occurrence of EC directional group selection provides an intuitive summary of the previously discussed parameters. If EC migration in wound healing was random (i.e., isotropic), one would expect 25% of the velocities to appear in the transverse region, 50% in the combined lateral region, and the remaining 25% in the reverse region. Control monolayers consistently displayed anisotropy with a bias in the transverse direction over the reverse direction (Figure 5e). Interestingly, actin-disrupted conditions did not appear to affect the directional anisotropy, while nocodazole-treated monolayers appeared to have a more isotropic distribution of directional grouping (Figure 5e).

These results suggest that actin and tubulin dynamics contribute little to the directional organization of persistence; however, overall persistence is affected by each perturbation, so both actin and tubulin dynamics contribute greatly to the overall persistence of EC migration. Additionally, these results indicate that actin and tubulin dynamics affects EC migration in different directions, as can be observed in (Figure 4 and Figure 5b–e). Interestingly, actin disruptions did little to impact mean speed and displacement in all directions. Tubulin disruptions, however, greatly affected EC speed and displacement; the directional organization of speed and displacement were also lost. These observations imply that other molecular mechanisms may contribute to regulating turning angle and persistence sensitivity to the direction.

### 3.3. Cytoskeletal Dynamics and Cell Morphology

**Figure 6:**
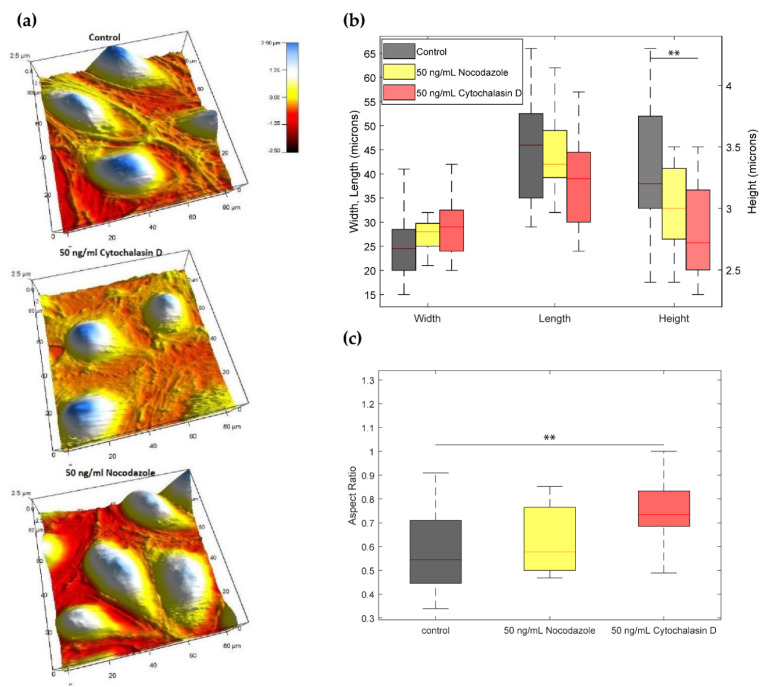
(**a**) Topographical images of cells taken with the Atomic Force Microscope. Cytochalasin D and nocodazole treatments were administered to cells four hours before imaging. (**b**) Measurements of length, width, and height from topographical images. (**c**) Aspect Ratio (width/length) calculations from width and length measurements of HUVECs. Within each box in the box and whisker plots, the horizontal lines denote median values; boxes extend from the 25th to the 75th percentile of each group’s distribution of values. Comparisons were carried out with a one-way ANOVA test. *p*-values were then calculated with a multi-comparison test (** *p* < 0.01).

To understand how actin and tubulin dynamics affected EC morphology, a high resolution non-optical imaging technique called AFM was used to interrogate cell topography and stiffness. From (Figure 6a), one can observe the topography of the control, actin, and tubulin-disrupted groups. Following from (Figure 6a), the height is defined as the point on the cell surface farthest away from its adherent surface. On the adherent surface, one can roughly approximate the EC basal contour with an oval. The largest diameter in this oval is the “length”, and the smallest diameter corresponds to the “width” of the EC.

The average height of the control ECs was 3.33 μm, compared to 2.82 μm and 3.05 μm for actin and tubulin-disrupted cells, respectively (Figure 6b). These averages were compared by one-way ANOVA with Tukey adjustment of *p*-values for multiple comparisons, which showed a significant difference between the control and the actin-disrupted cells (*p* < 0.01), while the tubulin-disrupted cells showed no difference with the control (*p* = 0.20). This suggests that the actin network is involved in regulating cell height.

The average widths of the control (25.00 μm), the actin-disrupted (28.88 μm), and tubulin-disrupted EC’s (27.07 μm) were not found to be significantly different (one-way ANOVA, *p* = 0.15). Similarly, the average lengths the control (45.31 μm), the actin-disrupted (38.31 μm), and tubulin-disrupted EC’s (44.00 μm) were not found to be significantly different (one-way ANOVA, *p* = 0.093).

Interestingly, even though there is no difference in mean widths and lengths between the three treatments, there is a difference in the median aspect ratio, defined as width/length (Figure 6c). The median aspect ratios for the control (0.54), the actin-disrupted (0.73), and tubulin-disrupted EC’s (0.58) were compared first by the Kruskal–Willis rank sum test and the overall *p* < 0.01 indicated a difference in the medians. The Dunn test with Bonferroni adjustment for multiple comparisons then showed that the median aspect ratios for actin-disrupted HUVECs and control are significantly different (*p* < 0.01). The median aspect ratio of tubulin-disrupted ECs showed no difference with either the control or actin-disrupted ECs (*p* = 0.16 and 0.22, respectively). These observations suggest that actin dynamics regulates EC height and aspect ratio.

### 3.4. Cytoskeletal Dynamics and Cell Stiffness

**Figure 7:**
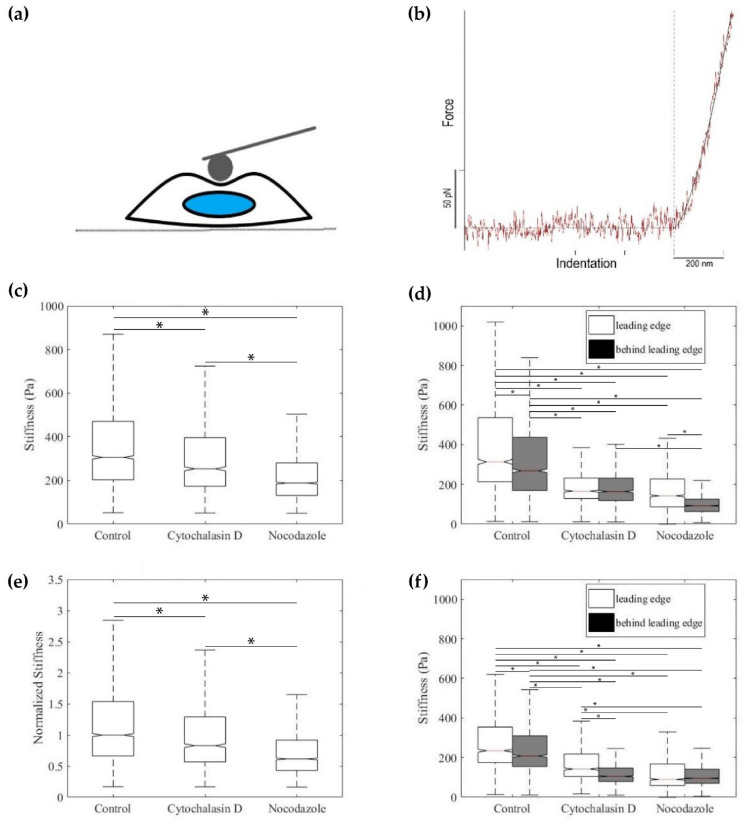
(**a**) Schematic of live cell indentation via AFM. (**b**) Force curve obtained over the duration of indentation. (**c**–**f**) The middle bar represents the median value of the distribution, and the notch represents the 95% confidence interval. (**c**) Compressive Modulus of ECs under varying cytoskeletal disruptions. (**d**) Measured compressive moduli of HUVECs 1 h after wound. (**e**) Compressive moduli of cytoskeletal disruptions normalized to the compressive modulus of the control. (**f**) Measured Compressive moduli 16 h after wound (* *p <* 0.05).

To understand the effect of actin and tubulin dynamics on EC stiffness, either actin or tubulin dynamics were disrupted in EC monolayers four hours before stiffness measurements via AFM. As seen in (Figure 7c), the compressive modulus was significantly lower after cells were exposed to 50 ng/mL cytochalasin D 253 Pa ± 12 Pa than the control group 306 Pa ±10 Pa (*p* < 0.05). Likewise, the compressive modulus of cells treated with 50 ng/mL nocodazole for four hours was significantly 188 Pa ± 7 Pa lower than the controls (*p* < 0.05), showing that actin and tubulin dynamics affect EC elasticity.

To see if there was an organization of EC stiffness and distance to the wound boundary, we prepared wound healing assays for AFM stiffness measurements (Figure 7e,f). Likewise, actin and tubulin disruptions were used to understand the effect of actin and tubulin dynamics on EC stiffness in wound healing models. Measurements after the first hour of the initial wound showed that ECs within 300 μm of the leading edge (the trailing region) were significantly stiffer than ECs beyond 300 μm of the leading edge (Figure 7e, *p* < 0.05). After the first hour of the initial wound, actin-disrupted monolayers did not display a difference in compressive modulus between the leading edge and trailing monolayer. Tubulin-disrupted monolayers, however, did display a significantly higher compressive modulus of cells closest to the leading edge compared to cells in the trailing region (*p* < 0.05). As observed previously, the control ECs at the leading edge were significantly stiffer than both actin and tubulin-disrupted ECs at the leading edge (*p* < 0.05). Likewise, after the first hour, control ECs in the trailing region had a significantly higher compressive modulus than the trailing region in actin and tubulin-disrupted ECs.

In addition to measuring ECs after the first hour of initial wounding, we also measured ECs 16 h after initial wounding, allowing us to see what happens to this organization of EC stiffness over time. After 16 h, the control maintained a significantly higher compressive modulus at the leading edge compared to the trailing region (Figure 7f, *p* < 0.05). This time, actin-disrupted ECs displayed a significantly higher stiffness at the leading edge than at the trailing region (Figure 7f, *p* < 0.05), but the tubulin-disrupted monolayer lost its difference in stiffness between the leading edge and trailing region (Figure 7e). Similar to measurements made one hour after the scratch, the control monolayer maintained a significantly higher stiffness at the leading edge compared to both actin and tubulin-disrupted monolayer stiffnesses at the leading edge (*p* < 0.05). At the trailing region, control EC stiffness was significantly higher than both actin and tubulin-disrupted monolayers (*p* < 0.05).

## 4. Discussion

Endothelial cells are intricately connected to their external environment through the cytoskeleton. Internal and external physical forces are transmitted through the cytoskeleton and affects cell mechanics and behavior [10,20,21,22,23]. Our studies show that in a wound healing environment, ECs regulate their speed and persistence in relation to the leading edge to efficiently migrate. Higher speed and persistence in the transverse direction allow ECs to capably migrate across the wound, and lower speed and persistence in the reverse direction enables ECs to quickly redirect their trajectory to the transverse zone with or without transitioning through the lateral zone. It is well known in the literature that individual cell turning corresponds to slower speeds [14], and the mathematical coupling between speed and persistence has been reported in this work and others [24,25]. Furthermore, these findings point to other mechanisms in filling the role. For instance, it is well established that Rho, Cdc42, Rac1, and phosphatidylinositol lipid (PtdIns) signaling systems regulate the randomness in cell migration and cytoskeletal dynamics [24,25,26].

At the specific concentration of cytochalasin D used in this study, we did not observe any effect on either mean speed or displacement nor was there an effect on the directional organization of mean speed and persistence. However, we did observe a decline in persistence for all directions, an effect on mean displacement organization and decline in gap closing speed compared to the control, suggesting that slightly disrupted actin dynamics already impair important migratory characteristics. The observed topographical images via AFM agree with cell spreading and migratory behavior on soft substrates [27,28,29]. The morphology observed in cytochalasin D-treated cells was consistent with the reported morphologies of cells with decreased stress fibers [28,29,30]. Disrupted collective behavior in actin-disrupted ECs may also be due to impaired mechanical coupling between actin stress fibers, cadherins, integrins, and highly dynamic actin behavior at both receptors [31].

Tubulin dynamics have been shown to regulate key structural and migratory characteristics in ECs [10,32]. Mean speed, mean displacement, and gap closing speed were all lowered after nocodazole treatment as per Figure 3 and Figure 4. Notably, overall persistence in all directions was lower in tubulin-disrupted groups, suggesting that tubulin dynamics contribute to a base level of persistence and directionality, consistent with the notion of tubulin’s role in determining cell polarity [29].

Interestingly, our results suggest a link between cytoskeletal dynamics, network integrity, and migration. AFM measurements suggest an alteration of EC elasticity as a result of actin and tubulin disruptions, with both cases showing lower moduli than the controls after four hours of disruption, consistent with studies on the disruption of the actin cytoskeleton [9]. A body of literature reports cell stiffening after nocodazole treatment [9,10,11,33]; however, these were measurements taken within one hour of nocodazole treatment. Subsequent works focusing on longer post-tubulin disruption periods reported a transient increase in cell stiffness, followed by a decrease in stiffness three hours after the initial 2 mM colchicine treatment [10]. Since the AFM measurements in this study focused on EC mechanics after 4 h of treatment, the reported findings are consistent with what is found in the literature [10]. The compressive moduli obtained with spherical tips reflect the global response of cell mechanics which may be lower than stiffnesses measured by sharp tips as reported in the literature [34,35]. In sum, results from the literature and this current work suggest that EC stiffening occurs shortly after microtubule disruption followed by cellular softening at four hours of sustained disruption. These observations may help characterize requirements for more robust predictive models, as shall be discussed.

Successful models of EC migration must account for the directional regulation of EC speed and persistence during collective migration. For example, the Ornstein–Uhlenbeck process, and modifications of it, is the simplest stochastic process that is used to describe persistent random walks [14], especially since the process assumes a base level of cellular persistence. Likewise, directional terms such as chemotaxis [14] may help explain the organization of speed and persistence in response to wound creation, but further investigation with these tools will be needed to better understand the efficacy of directional bias terms in modeling EC migration.

## 5. Materials and Methods

### 5.1. Cell Culture

Primary human umbilical vein endothelial cells (HUVECs) (passage 4–8) were maintained at 37 °C in 5% CO_2_ and 5% O_2_ on tissue culture flasks containing one of three types of media: Endothelial Medium (EM), Reduced Serum Medium (RSM), and Serum-Free Medium (SFM).

RSM consists of 95% Human Endothelial SFM (Gibco, Carlsbad, CA, USA), 2% fetal bovine serum (BioTC, Wayne, NJ, USA), 15 mM HEPES (Corning Inc, Corning, NY, USA), 50 μg/mL heparin (Sigma Aldrich, St. Louis, MO, USA), 1X Antibiotic/Antimycotic (Corning), 2 mM L-Glutamine (Corning), 250 ng/mL endothelial cell growth factor (Alfa-Aesar, Tewksbury, MA, USA), and 250 ng/mL fibroblast growth factor. For experiments, RSM was left as is or treated with 50 ng/mL cytochalasin D (MilliporeSigma, Burlington, MA, USA) or nocodazole (MilliporeSigma).

EM consists of 80% M199 basal medium (Corning), 20% fetal bovine serum (BioTC), 15 mM HEPES (Corning), 50 μg/mL heparin (Sigma Aldrich), 1X Penicillin/Streptomycin (Corning), 2 mM L-Glutamine (Corning), and 250 ng/mL endothelial cell growth factor (Alfa-Aesar).

Serum-Free Medium (SFM) consists of X-VIVO (Lonza, Lexington, MA, USA) supplemented with 1X antibiotic/antimycotic (Corning). All tests utilized ECs between passage 4 and 8.

### 5.2. Media Selection

While Fetal Bovine Serum (FBS) is commonly used in cell culture, it is commonly argued that more physiological vascular models should use little to no FBS [36]. To that end, we investigated the effect of culture media on the 2D wound healing model used in this study. The selected EC culture media formulations of varying FBS concentration are: EM—20%; RSM—2%; SFM—0%.

Endothelial Cells immersed either in EM or RSM were measured to have significantly higher gap closing speeds compared to SFM immersed monolayers with both conditions at least doubling gap closing speeds of monolayers immersed in SFM (see Supplementary Materials). The mean displacement speeds of RSM immersed cells are close in value to EM immersed cells (Supplementary Materials). For the case of EM and RSM monolayers, the respective mean displacement speeds were significantly higher than the calculated mean displacement speed of the SFM condition (*p* < 0.05). To facilitate a more physiological testing environment, and to minimize any interference between pharmacological disruptions and FBS, RSM was selected as the media to use in the subsequent tests. For more discussion on media selection, please see the Supplementary Materials.

### 5.3. Pharmacological Disruptions

About three to four hours prior to experimental observation, experimental groups were given either RSM treated with a final concentration of 50 ng/mL cytochalasin D to disrupt microfilament polymerization or with RSM treated with a final concentration of 50 ng/mL nocodazole to disrupt microtubule polymerization. Experimental groups were compared with control groups fortified with untreated RSM. As a note, we did not attempt a simultaneous disruption of cytoskeletal elements over concerns for EC viability in our assays.

### 5.4. Wound Healing Assay

The amount 2 × 10^4^ cells/cm^2^ were seeded onto a gelatin-coated polystyrene six-well plate containing RSM. Cells were incubated for at least 24 h at standard culture conditions before the experiment. The monolayers were then scratched with a 200 μL micropipette tip. The samples were mounted onto the incubating stage of the Zeiss AxioObserver and let sit in the incubating unit for three hours prior to time-lapse imaging.

### 5.5. Time-Lapse Microscopy

Samples were incubated on the stage of a Zeiss AxioObserver with a motorized and incubated stage under normal culture conditions for three hours before imaging to attenuate thermal drift. Individual regions were selected under the Tiles function, and images were taken at three-minute time intervals.

### 5.6. Track Quantification

Motile HUVECs were tracked and quantified by the FIJI plugin TrackMate [37]. Before tracking, relevant data on image dimensions and frame to frame time steps are loaded into the properties section of FIJI (Image→Properties), and the desired video is cropped around the edges and the background is filtered to reduce non-specific spot identification. After inputting the relevant parameters, the program is set to run and positional data is obtained. The resulting tracks represent cell trajectories sampled uniformly over the entire field of view (1750 μm × 1300 μm).

### 5.7. Atomic Force Microscopy

An AFM MFP-3D-Bio Atomic Force Microscope (Asylum Research, Santa Barbara, CA, USA) was used to probe the intrinsic cellular mechanical properties of HUVECs. Cells were plated at a density of 100,000 cells/mL on gelatin-coated BD Falcon Petri dishes (BD Biosciences, San Jose, CA, USA) 24 h before AFM experiments. During all measurements, live samples were maintained at 37 °C using an AFM Petri dish heater (Asylum Research, Santa Barbara, CA, USA).

### 5.8. Topography Measurement

AFM topography images (see Figure 6a) were obtained in contact mode using triangular silicon nitride probes (TR400PB Asylum Research Probes, Santa Barbara) with a nominal spring constant k = 0.09 N/m, and a scanning frequency and cantilever deflection set point of 1 Hz and 1 V, respectively, over an 80 μm×80 μm square. Aspect ratios were calculated with the cell width w (the shorter EC bulge in the adhesion plane) and the cell length l (the longer bulge in the adhesion plane) with the formula Aspect Ratio=w/l.

### 5.9. Elasticity Measurement

Compressive moduli of live HUVEC monolayers were obtained using a silicon nitride cantilever (Novascan, Ames, IA, USA) with a spherical borosilicate glass probe 5 μm in diameter. The probe was then calibrated to ensure its spring constant (k = 0.05 N/m) was consistent with the nominal stiffness provided by the manufacturer (k = 0.06 N/m). Cell monolayers were positioned under the AFM tip and force versus displacement curves were captured on a 32 × 32 grid spanning 60 × 60 μm (an area typically including 6–7 cells). Each force curve was recorded at a rate of 4 μm/s and indented a maximum distance of 200 nm. Curves with an undefined contact point or non-monotonic slope were rejected, consisting of less than 1% of the total force curves collected in the experiment. The Hertz-Sneddon (HS) model was used to fit the indentation using the AFM software [38]. To obtain the stiffness, we fit the curves to the force versus indentation relation *F* = 4/3[*E*/(1 − ν^2^)]*R*^1/2^ *δ*^3/2^, for the force (F), indentation (δ), given indentation curvature radius (R), and estimated Poisson’s ratio (ν = 0.45). We restricted the stiffness analysis to the cell body, thus excluding the substrate contributions in the cell periphery, in accordance with previous studies [34].

## 6. Conclusions

Endothelial Cells organize both speed and persistence at and beyond the leading edge which allows for straighter trajectories and boosted speeds across the wound. When moving in the reverse region, ECs reduce speed and enable more turning to attenuate erroneous displacements and bolster course correction. Coordinated sheet migration is regulated by actin and tubulin dynamics, hence affecting wound closing performance. The framework provided robust tools for analyzing single cell migration. Furthermore, these tools connect individual behavior to collective characteristics. Specifically, disruption of cytoskeletal elements was shown to affect overall persistence, the directional organization of speed, along with cell shape, and compressive moduli. Interestingly, insights from tubulin-disrupted ECs suggest that the directional organization of persistence and speed are not necessarily controlled by the same mechanism. Moreover, the reduction in cell compressive elasticity, combined with cell-shape deformation, indicate that actin and tubulin cytoskeleton act as a load-bearing network. Hence, cytoskeletal self-assembly and reorganization is vital in maintaining cell shape and stiffness. Together, the model and experimental measurements pave the way to decipher the role of biomechanics in cell migration and proliferation in angiogenesis.

## Data Availability

Data is contained within the article or Supplementary Materials.

## Supplementary Materials

The following are available online at https://www.mdpi.com/article/10.3390/ijms23010568/s1.

## References

[1] Costa G., Harrington K.I., Lovegrove H.E., Page D.J., Chakravartula S., Bentley K., Herbert S.P. (2016). Asymmetric division coordinates collective cell migration in angiogenesis. Nat. Cell Biol..

[2] Lauffenburger D.A., Horwitz A.F. (1996). Cell migration: A physically integrated molecular process. Cell.

[3] Binny R.N., Plank M.J., James A. (2015). Spatial moment dynamics for collective cell movement incorporating a neighbour-dependent directional bias. J. R. Soc. Interface.

[4] Li D., Wang Y.L. (2018). Coordination of cell migration mediated by site-dependent cell-cell contact. Proc. Natl. Acad. Sci. USA.

[5] Bui J., Conway D.E., Heise R.L., Weinberg S.H. (2019). Mechanochemical Coupling and Junctional Forces during Collective Cell Migration. Biophys. J..

[6] Michaelis U.R. (2014). Mechanisms of endothelial cell migration. Cell. Mol. Life Sci..

[7] Reinhart-King C.A., Dembo M., Hammer D.A. (2008). Cell-cell mechanical communication through compliant substrates. Biophys. J..

[8] Rotsch C., Radmacher M. (2000). Drug-induced changes of cytoskeletal structure and mechanics in fibroblasts: An atomic force microscopy study. Biophys. J..

[9] Borin D., Puzzi L., Martinelli V., Cibinel M., Lapasin R., Sbaizero O. (2017). An engineering insight into the relationship of selective cytoskeletal impairment and biomechanics of HeLa cells. Micron.

[10] Weber A., Iturri J., Benitez R., Zemljic-Jokhadar S., Toca-Herrera J.L. (2019). Microtubule disruption changes endothelial cell mechanics and adhesion. Sci. Rep..

[11] Wu H.W., Kuhn T., Moy V.T. (1998). Mechanical properties of L929 cells measured by atomic force microscopy: Effects of anticytoskeletal drugs and membrane crosslinking. Scanning.

[12] Charnick S.B., Lauffenburger D.A. (1990). Mathematical analysis of cell-target encounter rates in three dimensions. Effect of chemotaxis. Biophys. J..

[13] Douglas A., Lauffenburger J.J.L. (1993). Receptors: Models for Binding, Trafficking, and Signaling.

[14] Stokes C.L., Lauffenburger D.A., Williams S.K. (1991). Migration of individual microvessel endothelial cells: Stochastic model and parameter measurement. J. Cell Sci..

[15] Codling E.A., Plank M.J., Benhamou S. (2008). Random walk models in biology. J. R. Soc. Interface.

[16] Othmer H.G., Dunbar S.R., Alt W. (1988). Models of dispersal in biological systems. J. Math. Biol..

[17] Patlak C.S. (1953). Random walk with persistence and external bias. Bull. Math. Biophys..

[18] Uhlenbeck G.E., Ornstein L.S. (1930). On the Theory of the Brownian Motion. Phys. Rev..

[19] Kent J.T., Tyler D.E. (1988). Maximum likelihood estimation for the wrapped Cauchy distribution. J. Appl. Stat..

[20] Apontes P., Leontieva O.V., Demidenko Z.N., Li F., Blagosklonny M.V. (2011). Exploring long-term protection of normal human fibroblasts and epithelial cells from chemotherapy in cell culture. Oncotarget.

[21] Hayakawa K., Sato N., Obinata T. (2001). Dynamic reorientation of cultured cells and stress fibers under mechanical stress from periodic stretching. Exp. Cell Res..

[22] Ingber D.E., Wang N., Stamenovic D. (2014). Tensegrity, cellular biophysics, and the mechanics of living systems. Rep. Prog. Phys..

[23] Jaalouk D.E., Lammerding J. (2009). Mechanotransduction gone awry. Nat. Rev. Mol. Cell Biol..

[24] Maiuri P., Rupprecht J.F., Wieser S., Ruprecht V., Benichou O., Carpi N., Coppey M., De Beco S., Gov N., Heisenberg C.P. (2015). Actin flows mediate a universal coupling between cell speed and cell persistence. Cell.

[25] Verkhovsky A.B. (2015). The mechanisms of spatial and temporal patterning of cell-edge dynamics. Curr. Opin. Cell Biol..

[26] Arai Y., Shibata T., Matsuoka S., Sato M.J., Yanagida T., Ueda M. (2010). Self-organization of the phosphatidylinositol lipids signaling system for random cell migration. Proc. Natl. Acad. Sci. USA.

[27] Lo C.M., Wang H.B., Dembo M., Wang Y.L. (2000). Cell movement is guided by the rigidity of the substrate. Biophys. J..

[28] Raab M., Discher D.E. (2017). Matrix rigidity regulates microtubule network polarization in migration. Cytoskeleton.

[29] Discher D.E., Janmey P., Wang Y.L. (2005). Tissue cells feel and respond to the stiffness of their substrate. Science.

[30] Kumar S., Maxwell I.Z., Heisterkamp A., Polte T.R., Lele T.P., Salanga M., Mazur E., Ingber D.E. (2006). Viscoelastic retraction of single living stress fibers and its impact on cell shape, cytoskeletal organization, and extracellular matrix mechanics. Biophys. J..

[31] Collins C., Nelson W.J. (2015). Running with neighbors: Coordinating cell migration and cell-cell adhesion. Curr. Opin. Cell Biol..

[32] Kaverina I., Straube A. (2011). Regulation of cell migration by dynamic microtubules. Semin. Cell Dev. Biol..

[33] Zhou J., Kim H.Y., Wang J.H., Davidson L.A. (2010). Macroscopic stiffening of embryonic tissues via microtubules, RhoGEF and the assembly of contractile bundles of actomyosin. Development.

[34] Stroka K.M., Aranda-Espinoza H. (2011). Effects of Morphology vs. Cell-Cell Interactions on Endothelial Cell Stiffness. Cell. Mol. Bioeng..

[35] Vargas-Pinto R., Gong H., Vahabikashi A., Johnson M. (2013). The effect of the endothelial cell cortex on atomic force microscopy measurements. Biophys. J..

[36] van der Valk J., Bieback K., Buta C., Cochrane B., Dirks W.G., Fu J., Hickman J.J., Hohensee C., Kolar R., Liebsch M. (2018). Fetal Bovine Serum (FBS): Past-Present-Future. Altex.

[37] Tinevez J.Y., Perry N., Schindelin J., Hoopes G.M., Reynolds G.D., Laplantine E., Bednarek S.Y., Shorte S.L., Eliceiri K.W. (2017). TrackMate: An open and extensible platform for single-particle tracking. Methods.

[38] Dimitriadis E.K., Horkay F., Maresca J., Kachar B., Chadwick R.S. (2002). Determination of Elastic Moduli of Thin Layers of Soft Material Using the Atomic Force Microscope. Biophys. J..

