# Supplementary material for: Tracking of Endothelial Cell Migration and Stiffness Measurements Reveal the Role of Cytoskeletal Dynamics
Source: Int J Mol Sci. 2022 Jan 5;23(1):568. doi: 10.3390/ijms23010568 (PMC8745078; doi:10.3390/ijms23010568)
Supplement: Supplementary file 1 [file ijms-23-00568-s001.zip › ijms-1459895-supplementary.pdf]

A.

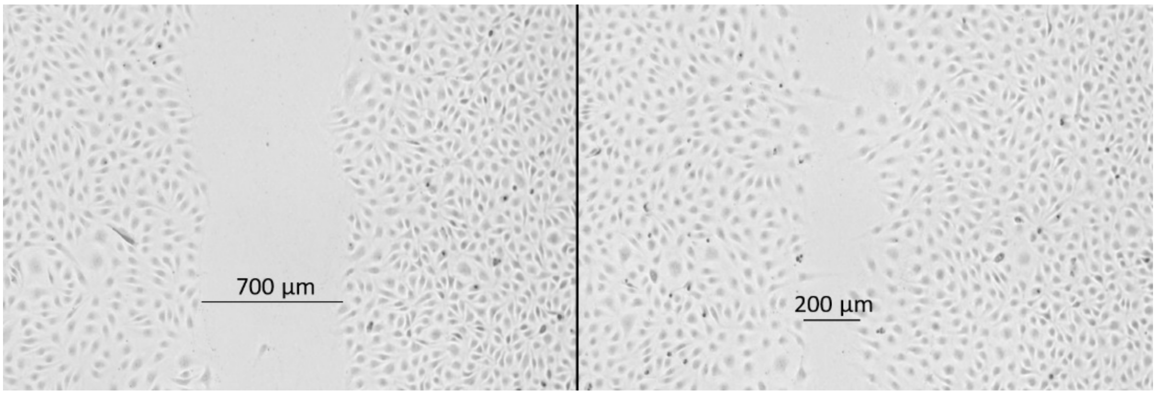

B.

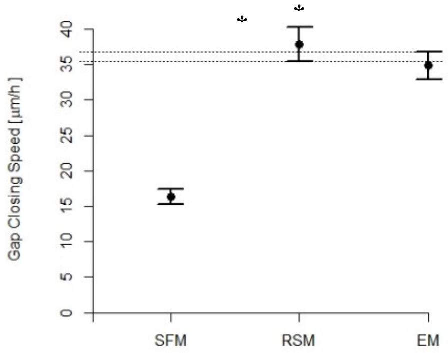

C.

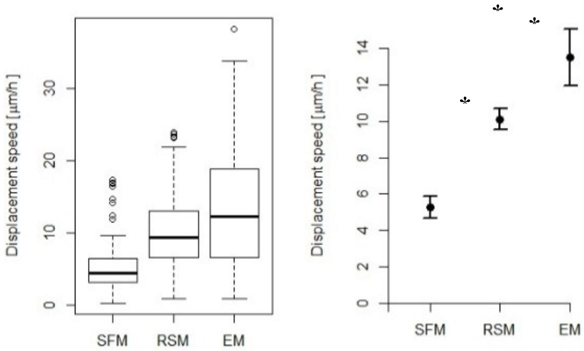

Figure S1. A. Representative demonstration of gap closing at initial time $t = 0$ (left), $t = 12h$ (right) for ECs in EM. **B.** Comparison of migration characteristics of cells immersed in media of varying FBS concentration (SFM, 0% FBS; RSM: Reduced Serum Medium, 2% FBS; EM: Endothelial Medium, 20% FBS). Gap Closure Speed is defined as the speed at which the two monolayer boundaries close their distance over time. **C.** Displacement speed is defined as the total displacement of the initial and final position of a cell over the total duration of the assay. (*: $p<0.05$, **: $p<0.01$)

Media affects the movement speed of cells. Time lapses of wound healing assays involving monolayers in media of varying FBS concentration were conducted to investigate a suitable minimum FBS concentration for more physiological media conditions. In wound healing, EM and RSM on average boast similar gap closing speeds. ECs immersed either in EM or RSM were measured to have significantly higher gap closing speeds compared to SFM immersed monolayers with both conditions at least doubling gap closing speeds of monolayers immersed in SFM (Fig. S-1B, $p<0.05$, Table S-IA).

Table S1. Calculated wound healing parameters based on time-lapse measurements of gap closure. **B.** Calculated wound healing parameters based on time-lapse measurements of gap closure.

**A.**	Replicate A			Replicate B			Replicate C		
	GCS[1] (μm/h)	SE[1] (μm/h)	Adjusted R^2	GCS[1] (μm/h)	SE**Error! Bookmark not defined.** (μm/h)	Adjusted R^2	GCS[1] (μm/h)	SE**Error! Bookmark not defined.** (μm/h)	Adjusted R^2
**EM[2]**	34.8	0.987	0.931	38.9	2.47	0.823	31.8	0.817	0.922
**RSM[2]**	37.8	1.22	0.916	35.8	1.75	0.896	22.8	0.641	0.920
**SFM[2]**	16.33	0.519	0.885	4.59	0.755	0.218	6.52	0.463	0.605
**B.**	**Replicate 1**			**Replicate 2**			**Replicate 3**		
	GCS[1] (μm/h)	SE**Error! Bookmark not defined.** (μm/h)	Adjusted R^2	GCS[1] (μm/h)	SE**Error! Bookmark not defined.** (μm/h)	Adjusted R^2	GCS[1] (μm/h)	SE**Error! Bookmark not defined.** (μm/h)	Adjusted R^2
**Control**	37.7	2.74	0.818	25.3	0.851	0.899	47.8	2.07	0.933
**CD[3]**	27.0	0.649	0.935	14.2	1.06	0.783	28.0	1.05	0.923
**Noc[3]**	12.7	0.572	0.783	9.33	0.825	0.721	18.3	0.982	0.839

The mean displacement speeds of RSM immersed cells are close in value to EM immersed cells (Fig. S-1B). The mean displacement speeds of EM and RSM monolayers were both significantly higher than the calculated mean displacement speed of the SFM condition ($p<0.05$).We wanted to investigate if there was some organization in the mean displacement speed of cells closer to the leading edge compared to cells farther from the leading edge. To do so, we measured the mean displacement speeds of the closest half of ECs to the leading edge, and the mean displacement speeds of the farthest half of ECs from the leading edge. Generally, ECs displayed a higher mean displacement speed for cells closer to the leading edge compared to cells farther than the leading edge (Table S-IIA). RSM immersed ECs displayed a significantly

[1] (GCS) — Gap Closing Speed. (SE) — Standard Error.
[2] (EM) — Endothelial Medium (20% Fetal Bovine Serum). (RSM) — Reduced Serum Medium (2% Fetal Bovine Serum). (SFM) — Serum Free Medium (No Fetal Bovine Serum)
[3] (CD) — 50ng/ml cytochalasin D. (Noc) — 50ng/ml nocodazole

higher mean displacement speed toward the leading edge compared to the trailing half (*p<0.001*). In most cases, SFM and EM immersed ECs maintained a significantly higher mean displacement speed for ECs closer to the leading edge compared to the half of ECs farther from the leading edge (*p<0.001*). To facilitate a more physiological testing environment, and to minimize any interference between pharmacological disruptions and FBS, RSM was selected as the media to use in the subsequent tests.

Table S2. Mean displacement speed grouped in terms of proximity to the leading edge.

A.

**Medium**	**Biological Replicate**	**HWD[4] (µm)**	**Mean 1st,4 (µm/h)**	**Mean 2nd,4 (µm/h)**	**p-value**
EM	A	386.7	12.73	14.02	0.4076
RSM	A	469.8	11.78	8.423	$8.50x10^{-9}$
SFM	B	491.3	6.294	4.239	$8.98x10^{-4}$
EM	B	558.2	9.042	7.328	0.005544
RSM	B	515.2	13.92	9.552	$1.43x10^{-13}$
SFM	B	395.0	4.766	4.938	0.8463
EM	C	616.5	10.44	5.456	$6.04x10^{-25}$
RSM	C	547.0	6.595	2.960	$2.86x10^{-56}$
SFM	C	569.0	3.631	2.051	$7.92x10^{-4}$

[4] (HWD) — Halfway distance between the leading edge and the field of view. (Mean 1st) — ECs between the leading edge and the halfway distance. (Mean 2nd) — ECs between the halfway distance and the field of view.

Table S3. Detailed migration parameter calculation for control and disrupted monolayers[5].

		Replicate 1			Replicate 2			Replicate 3		
		Control	CD	Noc.	Control	CD	Noc.	Control	CD	Noc.
Percent of cells in each direction[6]	Transverse	43.8	46.6	26.0	34.5	29.8	28.7	39.4	45.2	31.0
	Lateral	43.8	40.9	49.6	49.4	49.5	48.7	46.0	39.2	48.7
	Reverse	12.3	12.5	24.4	16.1	20.8	22.6	14.6	15.5	20.3
Speed by direction (μm/h)	Transverse	36.3	26.0	18.5	18.1	19.8	12.1	31.9	29.2	19.9
	Lateral	31.9	20.7	18.3	16.5	18.0	11.6	28.7	25.9	19.4
	Reverse	27.4	19.6	18.1	13.5	17.9	11.6	24.7	25.0	18.6
Maximum likelihood estimate ρ by direction	Transverse	0.72	0.61	0.36	0.51	0.43	0.29	0.60	0.54	0.31
	Lateral	0.62	0.42	0.32	0.40	0.34	0.22	0.46	0.29	0.22
	Reverse	0.49	0.19	0.33	0.21	0.29	0.17	0.35	0.09	0.1
Overall Estimated exponent μ^6 in t^μ		1.64	1.69	1.37	1.71	1.53	1.29	1.67	1.53	1.26
	NTG[6]	49.7	50.4	21.9	38.2	25.0	21.3	40.0	24.4	22.4
	n[6]	169	134	166	185	171	287	108	43	92

[5] For comparison purposes, all values were calculated based on the first ten hours of the experiment. Ten hours was selected as the shortest running time of the wound healing assays.

[6] The percentage of cells found on average in each region. ECs between the halfway distance and the field of view. μ is the exponent from the power law $MSD \sim t^\mu$, which is estimated through regression methods. The model is denoted as $MSD = at^\mu$, and in log-log coordinates, $\log(MSD) = \log(a) + \mu \log(t) \rightarrow y = \beta_0 + \mu x$. (NTG) — Net-to-Gross Percentage. This is calculated as the average of the ratio between the total displacement of ECs by their total path length, then multiplied by 100. (n) — Number of cell tracks sampled in each experiment.

Table S4. Statistical Comparison of EC ensemble speeds

Replicate	Comparison	*p*-value
1	Control-Cytochalasin D	0[7]
1	Control-Nocodazole	0[7]
1	Cytochalasin D-Nocodazole	0[7]
2	Control-Cytochalasin D	1.43E-71
2	Control-Nocodazole	0[7]
2	Cytochalasin D-Nocodazole	0[7]
3	Control-Cytochalasin D	6.09E-13
3	Control-Nocodazole	0[7]
3	Cytochalasin D-Nocodazole	3.56E-162

[7] We do not claim that the p value here is 0. The reported value is in fact a machine zero. For example, a double precision variable can display a minimum number n=2.225E-308. If the p-value calculation outputs a value p < n, then the machine will round p to 0. We claim that these values are so small that the machine cannot resolve them and are thus reported as 0.

Discussion into the Linear Fit of Gap Closing. When looking at the gap closing linear regressions, many readers will observe that some experiments will report an adjusted coefficient of variation (adjusted R^2) below 0.9 (for example, one experiment reported $R^2_{adj} = 0.748$). Assuming that R^2 is the sole metric that determines model performance, this may raise concerns among the readers about the model validity. However, we will assert that R^2_{adj} alone does not fully describe the performance of a model on a given dataset. This section will go further into the nuances of model choice, randomness within the data, and explaining the variation of data with respect to a chosen independent variable.

Firstly, the interpretation of R^2, R^2_{adj} is most precisely defined as the measure of variance of the dependent variable being studied is explained by the variance of the independent variable. For example, $R^2 = 0.245$ is read as "24.5% of the variation is explained by the model." For small or 'lower' R^2, concluding that the model poorly explains the data may be reasonable. However, careful considerations of other metrics and the data itself will provide a more comprehensive understanding of model validity on a given dataset.

For example, consider a dataset y sampled over time x. Let's say we are interested in modeling $y = f(x)$. For our purposes, let $f(x)$ be of the form

$$y = \beta_0 + \beta_1 x. \qquad (S1)$$

Let the *a priori* relationship between y and x is characterized as such,

$$y = ax + b + \mathcal{N}(\mu, \sigma^2). \qquad (S2)$$

Where a is the linear slope, b is a constant offset, and $\mathcal{N}(\mu, \sigma^2)$ is the normal distribution centered around the sample mean μ and σ^2 is the sample variance. Over an independent variable x, $\mathcal{N}(\mu, \sigma^2)$ may be characterized analytically with the following exponential,

$$\mathcal{N}(\mu, \sigma^2) = f(x \mid \mu, \sigma^2) = \frac{1}{\sigma\sqrt{2\pi}} e^{-\frac{1}{2}\left(\frac{x-\mu}{\sigma}\right)^2} \quad (S3)$$

In this case, let the random process fluctuate around a mean $\mu = 0$ such that we can refer to $\mathcal{N}(0, \sigma^2) \rightarrow \mathcal{N}(\sigma^2)$. Notice that as the variance is small ($\sigma^2 \rightarrow 0$), equation (S2) will approach the behavior of a linear equation. Looking at the behavior of large variance ($\sigma^2 \rightarrow \infty$), equation (S2) will approach the behavior of a normal distribution. Notice here that $\mathcal{N}(\sigma^2)$ is impossible to know in a dataset in practice and can be, at best, estimated. The best model to explain equation (S2) without accounting for the random fluctuations in the data is equation (S1). In the context of equation (S1), smaller variance within equation (S2) will be well described by (S1), while a more dominant random process will be poorly described by equation (S1), or

$$\lim_{\sigma^2 \rightarrow 0} R^2 \rightarrow 1$$

$$\lim_{\sigma^2 \rightarrow \infty} R^2 \rightarrow 0$$

However, even in the large variance limit, equation (S1) is still the best model for characterizing the relationship between y and x without attempts to estimate the random process. Applying this idea directly to the experimental observations, we simulated our gap closing data with equation (S2) with $\mu = 0$, or

$$D(t) = d_{o,gap} - v_{close}t + \mathcal{N}(\sigma^2) \quad (S4)$$

Based on the collected data, we select an initial gap size $d_0 = 600\mu m$, and gap closing speed $v_{close} = 30\mu m/h$ and freely vary σ^2 for the purposes of comparison. The results are illustrated in Figure S-2. As the variance increases, the simulated data transitions from an idealized linear equation ($\sigma^2 \rightarrow 0$) to the collected data in the scratch assay (Figure S-2). This result suggests that the average behavior of endothelial cells in 2D wound healing is linear with random

A

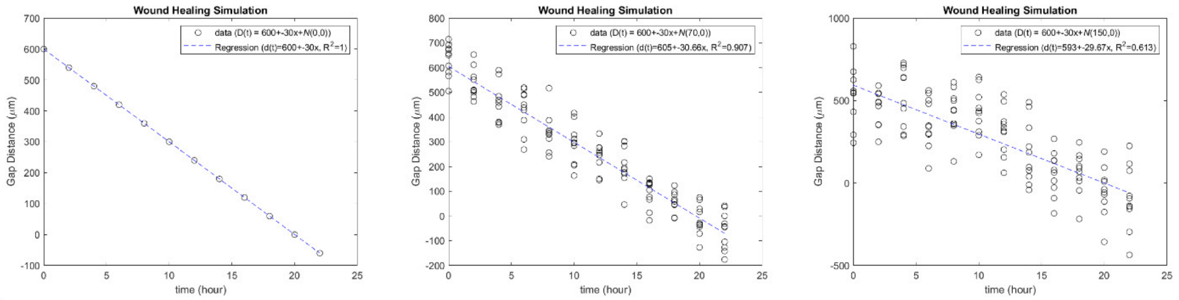

B

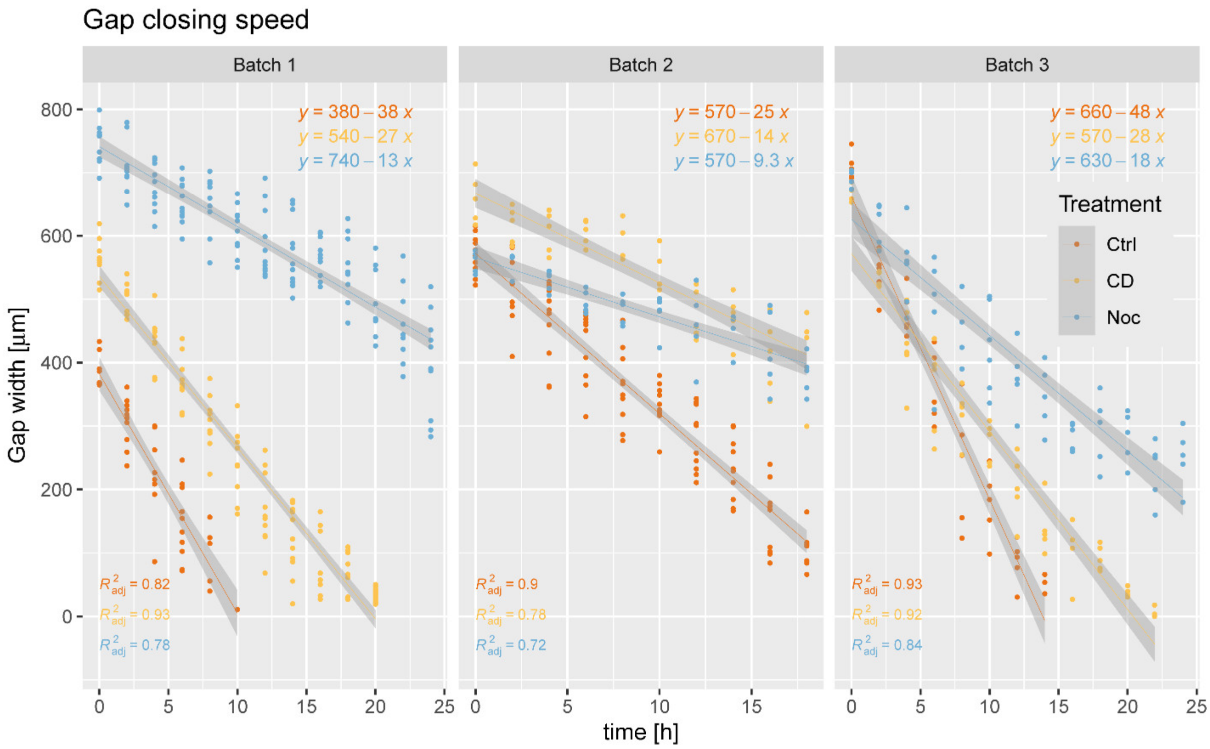

Figure S2. A. Wound healing simulation for varying noise spread. **B.** Real Gap Closing Data.

contributions over the contour position. Given that individual cell motion includes random motion, we think that the random fluctuations of the contour may be a feature of the individual cell characteristics.

Statistics for Figure Comparisons: Here we present the comparative statistics for Figures 3 to 5. As a preliminary remark, all error bars in the aforementioned figures represent the 95% confidence interval. If the error bars do not overlap, then there is less than a 5% chance that the mean values are indistinguishable. Larger distances between the confidence intervals indicate more distinct comparisons which correspond to lower p-values. The p-values are adjusted for multiple comparison by the Dunnett method unless otherwise specified.

Table S5. Statistical Comparison (*p*-value) for Figure 3(b)

Comparison	Replicate 1	Replicate 2	Replicate3
Control-CD	0.000197	1.25×10^{-14}	2.57×10^{-14}
Control-Noc	$< 2 \times 10^{-16}$	$< 2 \times 10^{-16}$	$< 2 \times 10^{-16}$

Table S6. Statistical Comparison (*p*-value) for Figure 3(d)

Comparison	Replicate 1	Replicate 2	Replicate3
Control-CD	5.68×10^{-9}	2.9×10^{-7}	0.414
Control-Noc	$< 10^{-10}$	$< 10^{-10}$	$< 10^{-10}$

Table S7. Statistics for Figure 4(a) Persistence parameter ρ by direction, lower and upper boundaries of the bootstrapped 95% confidence interval as presented in Figure 4(a). We also provide the lower and upper values of the 99% confidence interval for ρ. As a note, the 95% and 99% confidence intervals were calculated from separate bootstrapping runs.

Replicate	Treatment	Direction	rho	95% lower	95% upper	99% lower	99% upper
1	Control	Transverse	0.72	0.712	0.722	0.710	0.724
1	Control	Lateral	0.62	0.614	0.628	0.611	0.630
1	Control	Reverse	0.49	0.475	0.510	0.468	0.515
1	Cytochalasin D	Transverse	0.61	0.600	0.615	0.598	0.617
1	Cytochalasin D	Lateral	0.42	0.411	0.433	0.407	0.436
1	Cytochalasin D	Reverse	0.19	0.167	0.221	0.157	0.230
1	Nocodazole	Transverse	0.36	0.348	0.375	0.342	0.381
1	Nocodazole	Lateral	0.32	0.314	0.334	0.311	0.337
1	Nocodazole	Reverse	0.33	0.320	0.346	0.314	0.349
2	Control	Transverse	0.51	0.495	0.516	0.492	0.518
2	Control	Lateral	0.40	0.387	0.407	0.383	0.410
2	Control	Reverse	0.21	0.192	0.229	0.182	0.236
2	Cytochalasin D	Transverse	0.43	0.414	0.437	0.409	0.441
2	Cytochalasin D	Lateral	0.34	0.329	0.350	0.326	0.352
2	Cytochalasin D	Reverse	0.29	0.275	0.308	0.272	0.312
2	Nocodazole	Transverse	0.29	0.274	0.297	0.270	0.300
2	Nocodazole	Lateral	0.22	0.207	0.226	0.205	0.228
2	Nocodazole	Reverse	0.17	0.153	0.181	0.150	0.186
3	Control	Transverse	0.60	0.595	0.614	0.591	0.617
3	Control	Lateral	0.46	0.443	0.468	0.440	0.472
3	Control	Reverse	0.35	0.324	0.373	0.311	0.381
3	Cytochalasin D	Transverse	0.54	0.522	0.555	0.515	0.561
3	Cytochalasin D	Lateral	0.29	0.264	0.309	0.255	0.317
3	Cytochalasin D	Reverse	0.09	0.039	0.137	0.026	0.153
3	Nocodazole	Transverse	0.31	0.295	0.330	0.290	0.335
3	Nocodazole	Lateral	0.22	0.202	0.232	0.198	0.236
3	Nocodazole	Reverse	0.10	0.076	0.125	0.068	0.132

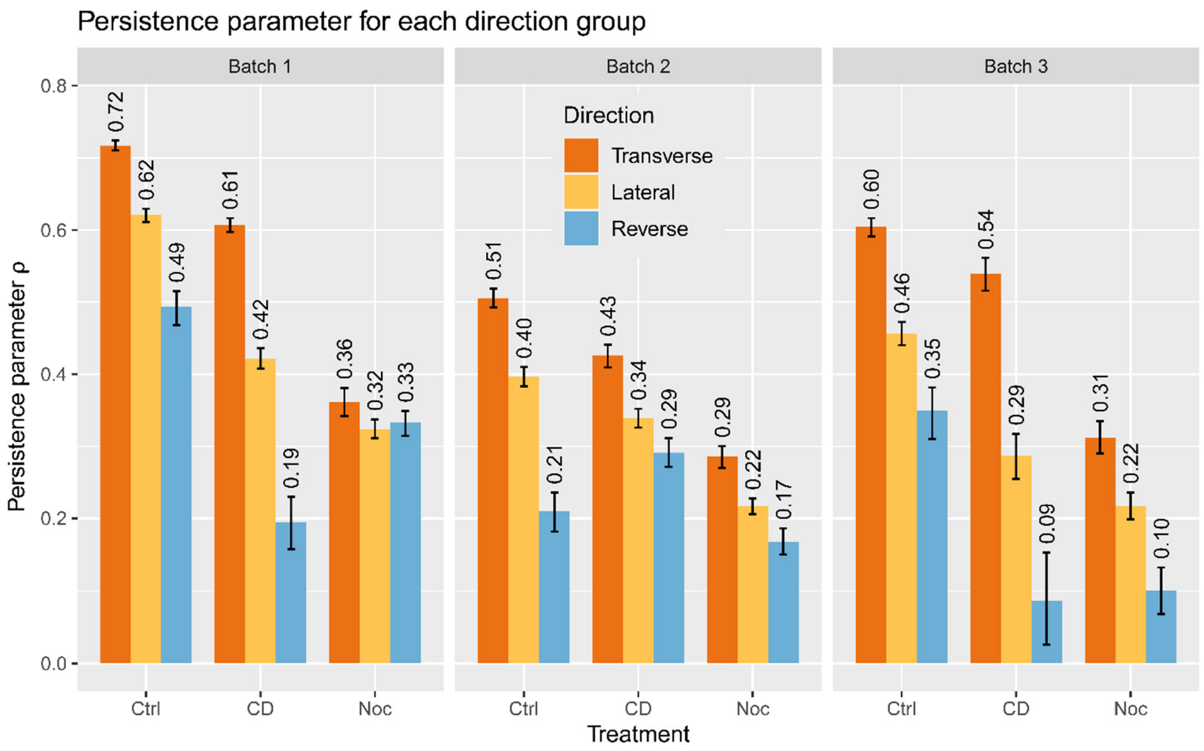

Figure S3. Persistence Parameter ρ by direction group. The error bars coorespond to the 99% confidence intervals. As such, non-overlapping intervals indicate a p-value boundary of $p <$ 0.01.

The estimate of ρ provides the shape parameter of the distribution of the turning angles. In a sense, it represents the concentration of turning angles around 0, or the idealized direction for each zone (Transverse, Lateral, and Reverse). The parameter ρ is obtained as the maximum likelihood estimate (MLE) of the distribution shape parameter for the turning angle. The MLE does not provide variability estimates from which we can obtain p-values. In light of this fact, we use the bootstrapping technique, a well-known statistical method, to estimate the variability. Bootstrapping is the process of obtaining the variability from one sample via resampling. We then estimate our parameter ρ for each resampled subset, and we then have a set of parameters ρ from which we can estimate the variability. In our case, we resampled each turning angle distribution 1000 times. From the empirical distribution of ρ obtain by bootstrapping, we can

calculate confidence interval for ρ at any level by using empirical quantiles. We remark that the bootstrapped confidence intervals do not come with p-values. In the paper, we provide 95% confidence intervals in Figure 4(a). To demonstrate that the ρ values are significant, we provide a calculation of the 99% confidence interval from a new bootstrapping run(Table S-VII). We additionally provide Figure S3, ρ by direction with 99% confidence intervals, to better illustrate the difference in ρ values.

Table S8. Statistics for Figure 4(b)

**Comparison**	**Replicate 1**			**Replicate 2**			**Replicate 3**		
	Control	CD	Noc.	Control	CD	Noc.	Control	CD	Noc.
Transverse-Lateral	<2e-16	<0.001	0.35	<2e-16	$< 10^{-6}$	$< 10^{-4}$	<2e-16	$< 10^{-4}$	0.14
Transverse-Reverse	<2e-16	<0.001	0.065	<2e-16	$< 10^{-6}$	0.0004	<2e-16	$< 10^{-4}$	0.0007
Lateral-Reverse	<2e-16	<0.001	0.46	<2e-16	0.936	0.9998	<2e-16	0.52	0.053

Note that the transverse, lateral, and reverse mean speeds for Nocodazole are close to each other. In Replicate 1 the mean speeds are 18.5, 18.3, and 18.1 $\mu m/h$ by direction. Likewise, the directional mean speeds are 12.1, 11.8, and 11.6 $\mu m/h$ respectively. In replicate 3, the directional mean speeds are 19.9, 19.4, and 18.6 $\mu m/h$ respectively. The standard error for the means is extremely small due to the large number of cell tracks and large number of time intervals (200) we have for each experiment. For example, there were 169 tracked cells and 200 time points in replicate 1, which results in 33,800 data points. Although there is a statistical difference between some of these values, there is no practical difference between them, especially in the first two replicates. Although the third replicate may have more pronounced differences in their means, the only notable difference is in the transverse-reverse comparison.

Table S9. Statistics for Figure 4(c)

Replicate	Treatment	n	Transverse (µm)	Lateral (µm)	pvalue
1	Control	153	155	73	1.92E-18
1	Cytochalasin D	121	116	41	5.04E-20
1	Nocodazole	86	32	24	0.0080
2	Control	151	58	37	1.44E-06
2	Cytochalasin D	124	41	26	0.00020
2	Nocodazole	198	20	14	5.43E-06
3	Control	91	118	47	5.30E-12
3	Cytochalasin D	39	106	30	2.07E-08
3	Nocodazole	73	37	22	2.01E-05

We note that the mean coordinate displacements in the transverse and lateral direction were performed by the paired t-test

Table S10. Number of reverse and transverse track displacements in 10 hours. Inconsistent reverse coordinate displacement numbers (ranging from 4 to 80) proved tough to fairly compare with the transverse and lateral coordinate displacements. This was one of the reasons as to why we excluded from the visualization in 4(c)

Replicate	Treatment	Direction	n()
1	Control	Transverse	153
1	Control	Reverse	16
1	Cytochalasin D	Transverse	121
1	Cytochalasin D	Reverse	13
1	Nocodazole	Transverse	86
1	Nocodazole	Reverse	80
2	Control	Transverse	151
2	Control	Reverse	34
2	Cytochalasin D	Transverse	124
2	Cytochalasin D	Reverse	47
2	Nocodazole	Transverse	198
2	Nocodazole	Reverse	89
3	Control	Transverse	91
3	Control	Reverse	17
3	Cytochalasin D	Transverse	39
3	Cytochalasin D	Reverse	4
3	Nocodazole	Transverse	73
3	Nocodazole	Reverse	19

Table S11. Statistics for Figure 4(d). All p-values are comparisons for the front vs. back cells.

Comparison	Replicate 1	Replicate 2	Replicate3
Control	0.0178	$< 10^{-6}$	$< 10^{-5}$
Cytochalasin D	0.00042	0.00083	$< 10^{-6}$
**Nocodazole**	0.00526	0.00011	$< 10^{-6}$

Table S12. Statistics for Figure 4(e). All p-values are comparisons for the front vs. back cells.

Comparison	Replicate 1	Replicate 2	Replicate3
Control	0.0083	$< 10^{-6}$	$< 10^{-6}$
Cytochalasin D	0.00012	$< 10^{-5}$	$< 10^{-4}$
**Nocodazole**	0.00038	$< 10^{-5}$	$< 10^{-5}$

Table S13. Statistics for Figure 4(f). All p-values are comparisons for the front vs. back cells.

Comparison	Replicate 1	Replicate 2	Replicate3
Control	0.069	0.00525	0.137
Cytochalasin D	0.375	0.355	0.180
**Nocodazole**	0.169	0.454	0.0098

Table S14. Statistics for Figure 5(d). General persistence parameter μ from log-log regression. We also provide the error and 95% confidence interval boundaries for the slope. The Slope p-value provides a confidence measure for the estimate, the lower the better. Additionally, we compared the differences in slopes for each batch by running a regression with the factor for treatment (Cytochalasin and Nocodazole). When comparing the slopes for the control and treatment, we found significance with $p < 10^{-16}$ for all cases.

Replicate	Treatment	Slope (µ)	Slope Error	95% Lower	95% Upper	Slope p-value	Adjusted R Squared
1	Control	1.64	0.0037	1.630	1.645	1.87E-299	0.999
1	Cytochalasin D	1.69	0.0038	1.683	1.698	4.03E-298	0.999
1	Nocodazole	1.37	0.0032	1.363	1.375	1.69E-295	0.999
2	Control	1.71	0.0037	1.700	1.715	2.31E-302	0.999
2	Cytochalasin D	1.53	0.0028	1.523	1.535	2.72e-315	0.999
2	Nocodazole	1.29	0.0038	1.280	1.295	7.75E-275	0.998
3	Control	1.67	0.0047	1.660	1.679	5.33E-280	0.998
3	Cytochalasin D	1.53	0.0072	1.513	1.541	7.88E-236	0.996
3	Nocodazole	1.26	0.0048	1.254	1.273	1.31E-253	0.997
